# Protein O-GlcNAcylation Promotes Trophoblast Differentiation at Implantation

**DOI:** 10.3390/cells9102246

**Published:** 2020-10-06

**Authors:** Peter T. Ruane, Cheryl M. J. Tan, Daman J. Adlam, Susan J. Kimber, Daniel R. Brison, John D. Aplin, Melissa Westwood

**Affiliations:** 1 Maternal and Fetal Health Research Centre, Division of Developmental Biology and Medicine, School of Medical Sciences, Faculty of Biology, Medicine and Health, University of Manchester, St. Mary’s Hospital, Manchester M13 9WL, UK; cheryl-mj.tan@cardiov.ox.ac.uk (C.M.J.T.); daman.adlam@manchester.ac.uk (D.J.A.); daniel.brison@manchester.ac.uk (D.R.B.); john.aplin@manchester.ac.uk (J.D.A.); melissa.westwood@manchester.ac.uk (M.W.); 2 Maternal and Fetal Health Research Centre, Manchester University NHS Foundation Trust, Manchester Academic Health Sciences Centre, Manchester M13 9WL, UK; 3 Division of Cell Matrix Biology and Regenerative Medicine, School of Biological Sciences, Faculty of Biology Medicine and Health, University of Manchester, Michael Smith Building, Manchester M13 9PT, UK; sue.kimber@manchester.ac.uk; 4 Department of Reproductive Medicine, Old St. Mary’s Hospital, Manchester University NHS Foundation Trust, Manchester Academic Health Science Centre, Oxford Road, Manchester M13 9WL, UK

**Keywords:** embryo implantation, stress, transcription factors, implantation failure, trophoblast differentiation, protein O-GlcNAcylation, extra-embryonic development

## Abstract

Embryo implantation begins with blastocyst trophectoderm (TE) attachment to the endometrial epithelium, followed by the breaching of this barrier by TE-derived trophoblast. Dynamic protein modification with O-linked β-N-acetylglucosamine (O-GlcNAcylation) is mediated by O-GlcNAc transferase and O-GlcNAcase (OGA), and couples cellular metabolism to stress adaptation. O-GlcNAcylation is essential for blastocyst formation, but whether there is a role for this system at implantation remains unexplored. Here, we used OGA inhibitor thiamet g (TMG) to induce raised levels of O-GlcNAcylation in mouse blastocysts and human trophoblast cells. In an in vitro embryo implantation model, TMG promoted mouse blastocyst breaching of the endometrial epithelium. TMG reduced expression of TE transcription factors *Cdx2*, *Gata2* and *Gata3*, suggesting that O-GlcNAcylation stimulated TE differentiation to invasive trophoblast. TMG upregulated transcription factors *OVOL1* and *GCM1*, and cell fusion gene *ERVFRD1*, in a cell line model of syncytiotrophoblast differentiation from human TE at implantation. Therefore O-GlcNAcylation is a conserved pathway capable of driving trophoblast differentiation. TE and trophoblast are sensitive to physical, chemical and nutritive stress, which can occur as a consequence of maternal pathophysiology or during assisted reproduction, and may lead to adverse neonatal outcomes and associated adult health risks. Further investigation of how O-GlcNAcylation regulates trophoblast populations arising at implantation is required to understand how peri-implantation stress affects reproductive outcomes.

## 1. Introduction

Pre-implantation embryo development culminates in the formation of a blastocyst consisting of three lineages: the epiblast and primitive endoderm of the inner cell mass and the outer trophectoderm (TE) [1]. The embryo implants as the TE attaches to the luminal epithelium of the endometrium, and TE-derived trophoblast invades the stroma and goes on to form the maternal interface of the placenta [2]. However, the transition from TE to trophoblast at implantation is ill-defined, despite the importance of the balanced formation of proliferative and terminally differentiated trophoblast populations to the development of a healthy placenta [3]. Moreover, it is important to understand the sensitivity of trophoblast development to increasingly prevalent assisted reproduction technologies (ART) and maternal pathologies such as diabetes, obesity and hypertension, as there is evidence that these can lead to placental phenotypes that impact fetal growth and thus long term offspring health [4,5,6,7,8].

Embryos use a variety of signalling pathways to sense environmental stressors and elicit responses enabling stress resolution or adaptation [9]. We have previously shown that in response to osmotic stress, the c-Jun N-terminal kinase (JNK) pathway triggers embryonic invasion of endometrial epithelium in vitro, implicating stress signalling in TE differentiation to trophoblast at implantation [10]. Moreover, JNK regulates trophoblast functions during placentation [11]. Embryos are acutely sensitive to nutrient imbalances or deprivation. Signalling through adenosine monophosphate-activated kinase (AMPK) and mammalian target of rapamycin (mTOR) mediates responses to nutritive stress in embryos [12,13], leading to increased endocytosis and autophagy [14,15], altered trophoblast differentiation [16], and ultimately offspring health consequences [17,18]. However, unlike these pathways which transduce signals through protein phosphorylation, post-translational modification with O-linked β-N-acetylglucosamine (O-GlcNAcylation) is recognised as an important cellular response to a wide range of environmental stressors, including osmotic, oxidative, temperature and genotoxic stresses [19]. The substrate for O-GlcNAcylation, uridine diphosphate-N-acetylglucosamine (UDP-GlcNAc) is produced by conjugation of glucose with amino acid-derived amine, lipid-derived acetyl and the nucleotide uridine in the hexosamine biosynthetic pathway (HBP). Cycling of O-GlcNAcylation at serine and threonine residues is mediated by a single pair of enzymes—O-GlcNAc transferase (OGT) and O-GlcNAcase (OGA)—and serves to regulate protein function both independently and together with phosphorylation [20]. The HBP and O-GlcNAcylation are, therefore, considered to integrate environmental adaptation with nutrient availability [21]. Correspondingly, metabolic disruption and pathologies, including high fat and sugar diets, obesity and diabetes, are associated with elevated O-GlcNAcylation levels in many tissues [22,23,24].

Metabolite flux through the HBP is required for pre-implantation embryo development [25,26], with this pathway being the major sink for glucose at this stage [27]. Current evidence suggests that O-GlcNAcylation downstream of the HBP is required for embryonic genome activation and specification of the TE lineage, through regulation of nuclear localisation of tricarboxylic acid enzymes and Yes-associated protein 1 (YAP1), respectively [27,28]. However, a recent study reported that the increased O-GlcNAcylation observed in blastocysts and endometrium of a mouse model of diabetes is associated with reduced implantation [29]. Furthermore, placental O-GlcNAcylation is increased in response to stressors such as noise, restraint or disturbance during early gestation, giving rise to altered offspring growth and behaviour [30]. Increased O-GlcNAcylation due to OGA deletion leads to impaired vascularisation at the placental exchange interface, dramatically impacting placental function [31]. Nutrient and stress sensing through the HBP and O-GlcNAcylation appear to fine-tune placental development [32]; however, the impact of this pathway during the initial stages of trophoblast development at implantation is unknown. Here, we set out to assess the effects of O-GlcNAcylation on TE and trophoblast function at implantation.

## 2. Materials and Methods

### 2.1. Cell Culture

The human endometrial adenocarcinoma-derived Ishikawa cell line was obtained from ECACC (99040201). The human choriocarcinoma-derived BeWo trophoblast cell line was gratefully received from Dr Margaret Saunders (University of Bristol). Both cell lines were cultured in 1:1 DMEM:F12 (Sigma, Gillingham, UK) supplemented with 10% fetal bovine serum (Sigma), 2 mM L-glutamine, 100 µg/mL streptomycin and 100 IU/mL penicillin (Sigma) at 37 °C, 5% CO_2_.

### 2.2. Mouse Embryo Collection

Mouse embryos were collected under UK Home Office project license PPL 70/07838 (approved 16 October 2013), as authorised by the Animal Welfare and Ethical Review Board of the University of Manchester, according to the Animal Act, 1986. Eight-week-old CD1 female mice (Charles River, Portishead, UK) were injected (intraperitoneal) with 5 IU pregnant mare serum gonadotrophin (Intervet, Milton Keynes, UK) and, 46 h later, 5 IU human chorionic gonadotrophin (Intervet) to induce superovulation. Mice were then housed overnight with <9 month old CD1 male mice (Charles River). A total of 28 h after separation from males, oviducts were dissected from female mice and 2-cell embryos at embryonic day (E)1.5 were collected by flushing oviducts with M2 medium (Millipore, Watford, UK). Embryos were cultured in KSOM medium (Millipore) containing 0.4% bovine serum albumin (BSA, Sigma) under oil (Vitrolife, Warwick, UK) at 37 °C, 5% CO_2_ to the blastocyst stage at E4.5, before hatching from the zona pellucidae using acid Tyrode’s solution (pH 2.5) (Sigma) and incubation to E5.5 in 1:1 DMEM:F12 supplemented with 2 mM L-glutamine, 100 µg/mL streptomycin, 100 IU/mL penicillin and 0.4% BSA.

### 2.3. In Vitro Implantation Model Using Mouse Blastocysts

Ishikawa cells were cultured to confluency over 3–4 days in 24-well plates (Greiner, Stonehouse, UK) on 13 mm glass coverslips coated with 2% Matrigel (Sigma). Cells were incubated in serum-free medium 24 h prior to co-culture with three E5.5 blastocysts per well, and assessment of attachment stability over 48 h co-culture using an inverted phase contrast microscope (Evos XL Core, ThermoFisher, Loughborough, UK), as previously described [33]. Co-cultures were fixed after 48 h with phosphate-buffered saline (PBS)–4% paraformaldehyde (PFA) for 20 min at room temperature and stored under PBS at 4 °C.

### 2.4. Immunofluorescence Staining

Mouse blastocysts were fixed in PBS, 0.4% polyvinylpyrrolidone (PVP)–4% PFA for 20 min, quenched in PBS, 0.4% PVP-50 mM ammonium chloride for 5 min and permeabilised using PBS, 0.4% BSA–0.1% Triton-X100 for 5 min. Blastocysts were incubated overnight at 4 °C in PBS, 0.4% BSA containing primary antibody (Table 1) under mineral oil (Sigma), then washed and incubated at room temperature under oil in PBS, 0.4% BSA containing Alexa 488/649-conjugated anti-mouse/rabbit IgG antibody (Life Technologies, Inchinnan, UK), Alexa 568-conjugated phalloidin (Life Technologies) and 4′,6-diamidino-2-phenylindole (DAPI, Sigma) for 1 h. Embryos were imaged in PBS–3% 1,4-diazabicyclo [2.2.2]octane (DABCO, Sigma) in a glass bottom dish (Iwaki, Stone, UK).

Coverslips with mouse embryos attached to Ishikawa cells or with BeWo cells were fixed with PBS–4% PFA for 20 min, quenched for 5 min with PBS-50 mM ammonium chloride and permeabilized for 5 min in PBS–0.5% Triton-X100. Samples were then incubated with primary antibodies (Table 1) in PBS at room temperature for 2 h, before washing with PBS and room temperature incubation for 1 h with PBS containing Alexa 568-conjugated phalloidin and DAPI. Coverslips with mouse embryos attached to Ishikawa cells were mounted in a chamber of 3% DABCO in PBS for microscopy, and those with BeWo cells were mounted on glass slides in Mowiol 4–88 mounting medium (Sigma) containing 3% DABCO.

### 2.5. Fluorescence Microscopy

Images were captured with an Apotome 2-equipped Zeiss Axiophot microscope (Zeiss, Cambourne, UK) using Zen 2.0 software (Zeiss). Analysis was performed with Zen 2.0. Up to 30 optical sections of blastocysts were obtained at 4 µm increments, and those of blastocysts attached to Ishikawa cells were obtained at 2 µm increments. Single plane images of BeWo cells were acquired without the use of Apotome 2 module.

### 2.6. RNA Extraction and Quantitative PCR

RNA was extracted from blastocysts (10 per experiment) and from 6-well dishes of BeWo cells using the RNeasy Micro Kit (Qiagen, Manchester, UK). Reverse transcription (RT) reactions were carried out with 12 ng RNA, random 9mer primers (Agilent, Wokingham, UK) and a Sensiscript RT kit (Qiagen). Quantitative PCRs (qPCRs) were performed using the RT reactions, 0.25 μM primers (Table 2) and a QuantiTect SYBR green PCR kit (Qiagen). qPCR reactions were run on a Stratagene Mx3000p machine with thermocycling according to QuantiTect instructions (35 cycles using 58 °C annealing temperature for all primers). Stratgene MxPro analysis calculated cycle threshold (Ct) values which were used to determine expression relative to housekeeping genes (Table 2). Sample RNA-negative and reverse transcriptase-negative RT reactions were used as controls in qPCR reactions. Primer specificity was confirmed by performing dissociation curves in all qPCR runs.

### 2.7. Statistical Analysis

Quantified data are represented as the median ± interquartile range (IQR) or mean ± standard error of the mean (SEM). Mann–Whitney and Kruskal–Wallis tests were performed to assess statistical significance using Prism (GraphPad Software Inc., San Diego, CA, USA).

## 3. Results

### 3.1. TMG Increases O-GlcNAcylation and Alters OGT and OGA Localization in Mouse Blastocysts

**Figure 1:**
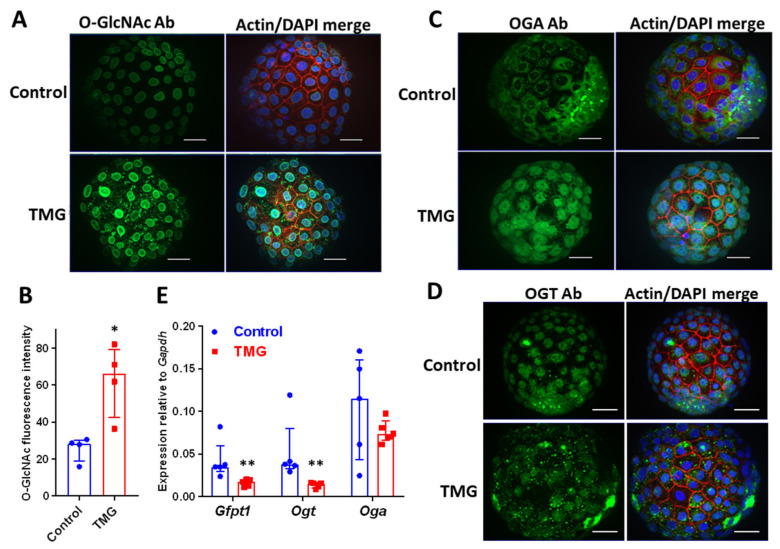
E4.5 mouse blastocysts were cultured for 24 h to E5.5 in the absence (control) or presence of 5 µM TMG. (**A**) Fixed blastocysts stained with phalloidin to label actin filaments (red), DAPI to label nuclei (blue) and RL2 anti-O-GlcNAcylated protein antibody (green). Fluorescence microscopy was performed with optical sectioning, and maximum intensity projections of optical sections are shown. Scale bars 20 µm. (**B**) Quantification of RL2 anti-O-GlcNAcylated protein antibody mean fluorescence intensity in blastocysts (arbitrary units) (*n* = 4 blastocysts from two independent experiments; median ± interquartile range (IQR); * *p* < 0.05 Control vs. TMG; Mann–Whitney). (**C**) Fixed blastocysts were stained with anti-OGA antibody (green) or (**D**) anti-OGT antibody (green), together with phalloidin (red) and DAPI (blue), and imaged as described. Maximum intensity projections are shown. Scale bars 20 µm. (**E**) RNA was extracted from groups of ten blastocysts and qPCR was performed to measure the expression of *Gfpt1*, *Ogt* and *Oga* (*n* = 5; median ± IQR; ** *p* < 0.01 Control vs. TMG; Mann–Whitney).

To increase O-GlcNAcylated protein levels, mouse blastocysts were treated with the OGA inhibitor thiamet g (TMG) at 5 µM [33,34,35] during the E4.5–5.5 period when embryos are sensitive to signals that activate invasive implantation [10,36,37]. Untreated mouse blastocysts exhibited O-GlcNAcylated protein staining in the nucleus and especially the nuclear membrane (Figure 1A). An increase in O-GlcNAc nuclear labelling and the formation of cytoplasmic puncta was observed in TMG-treated blastocysts (Figure 1A,B). OGT and OGA enzymes mediate dynamic O-GlcNAcylation, and in control blastocysts, their localization mirrored O-GlcNAc labelling; OGA was excluded from nuclei (Figure 1C) while OGT was localized predominantly to nuclei (Figure 1D). TMG treatment led to the concentration of OGA into nuclei (Figure 1C), whereas OGT localization did not change (Figure 1D). qPCR demonstrated that *Ogt* and the rate-limiting HBP enzyme, *Gfpt1*, were expressed at lower levels after TMG treatment, whereas *Oga* levels were not affected (Figure 1E). TMG did not affect gross blastocyst morphology.

### 3.2. TMG Treatment of Blastocysts Promotes Invasion in a Model of Implantation

**Figure 2:**
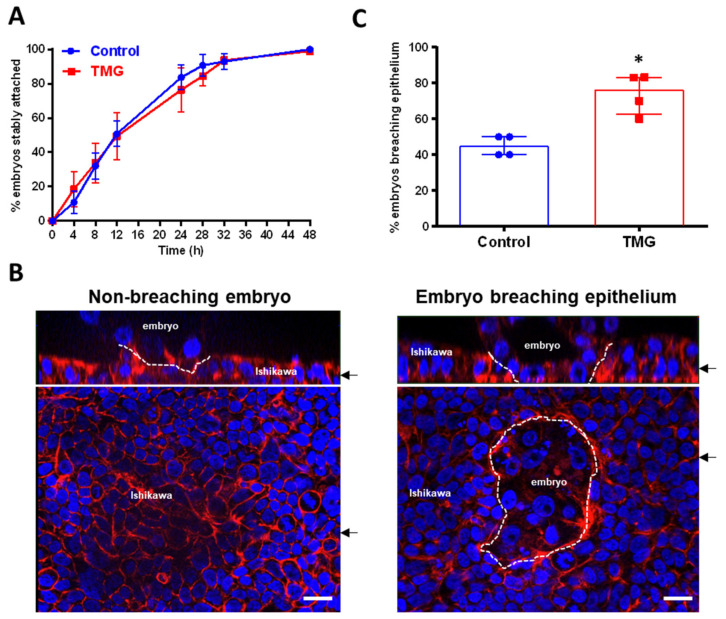
E5.5 blastocysts, previously untreated (control) or treated with 5 µM TMG were co-cultured with Ishikawa cell layers for 48h. (**A**) The percentage of stably attached embryos was monitored throughout co-culture (*n* = 4; 12–16 embryos per experiment; mean ± SEM). (**B**) Co-cultures were fixed after 48 h and stained with phalloidin to label actin (red) and DAPI to label nuclei (blue). Fluorescence microscopy with optical sectioning allowed for the determination of embryonic breaching of the Ishikawa cell layer. Dotted lines indicate embryo–Ishikawa interface. Single optical sections are shown in lower panels and Z-stacks of optical sections in upper panels. Arrows indicate position of optical section and Z-stack. Embryos and Ishikawa cell are labelled. Scale bars 20 µm. (**C**) Percentage embryos that have breached the Ishikawa cell layer (*n* = 4; 12–16 embryos per experiment; median ± IQR; * *p* < 0.05; Mann–Whitney test).

After 24 h exposure to control medium or TMG-containing medium, E5.5 blastocysts were co-cultured with Ishikawa cell layers to model embryo attachment and invasion of endometrial epithelium at implantation [36]. The kinetics of stable attachment did not differ between control and TMG-treated blastocysts over 48 h (Figure 2A). Blastocyst co-culture with Ishikawa cells from E4.5–5.5 promotes trophoblast giant cell (TGC) invasion of the Ishikawa cell layer (Figure 2B) [36]. As co-culture here began at E5.5, the majority of control blastocysts did not breach the Ishikawa cells (Figure 2B,C); however, TMG-treated blastocysts were significantly more invasive (Figure 2C).

### 3.3. TMG Alters TE Transcription Factor Expression in Mouse Blastocysts

**Figure 3:**
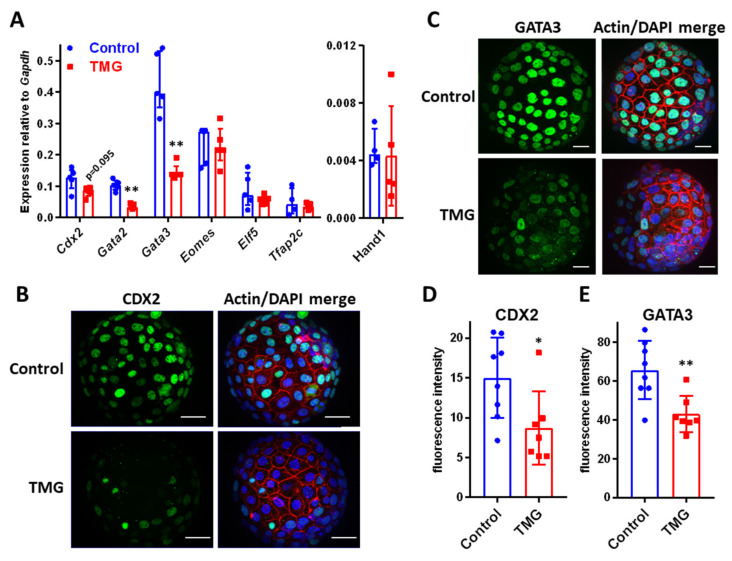
(**A**) qPCR was performed on groups of ten blastocysts to measure gene expression (*n* = 4–5; median ± IQR; ** *p*<0.01 Control vs. TMG; Mann–Whitney test). (**B**) Fixed blastocysts were stained with phalloidin (red), DAPI (blue) and anti-CDX2 antibody (green) or (**C**) anti-GATA3 antibody (green). Fluorescence microscopy was performed with optical sectioning, with maximum intensity projections shown. Scale bars 20 µm. (**D**) Quantification of anti-CDX2 and (**E**) anti-GATA3 antibody mean fluorescence intensity in blastocysts (arbitrary units). (*n* = 7–8 blastocysts from two independent experiments; median ± IQR; * *p* < 0.05 ** *p* < 0.01 Control vs. TMG; Mann–Whitney).

Our previous characterisation of this implantation model demonstrated that E4.5–5.5 co-cultured with Ishikawa cells alters TE transcription factor expression and leads to invasive TGC formation [36]. We, therefore, assessed the expression of six transcription factors associated with maintenance of TE phenotype in E5.5 blastocysts after 24 h treatment with control medium or TMG. *Gata2* and *Gata3* were significantly downregulated by TMG, while there was a trend towards downregulation of *Cdx2* (Figure 3A). Immunostaining demonstrated downregulation of CDX2 and GATA3 protein in TMG-treated blastocysts (Figure 3B–E). Hand1 is an early transcription factor in TGC differentiation; however, TMG did not increase *Hand1* expression (Figure 3A).

### 3.4. TMG Stimulates Syncytiotrophoblast Transcription Factor Expression in a Human Trophoblast Cell Line

**Figure 4:**
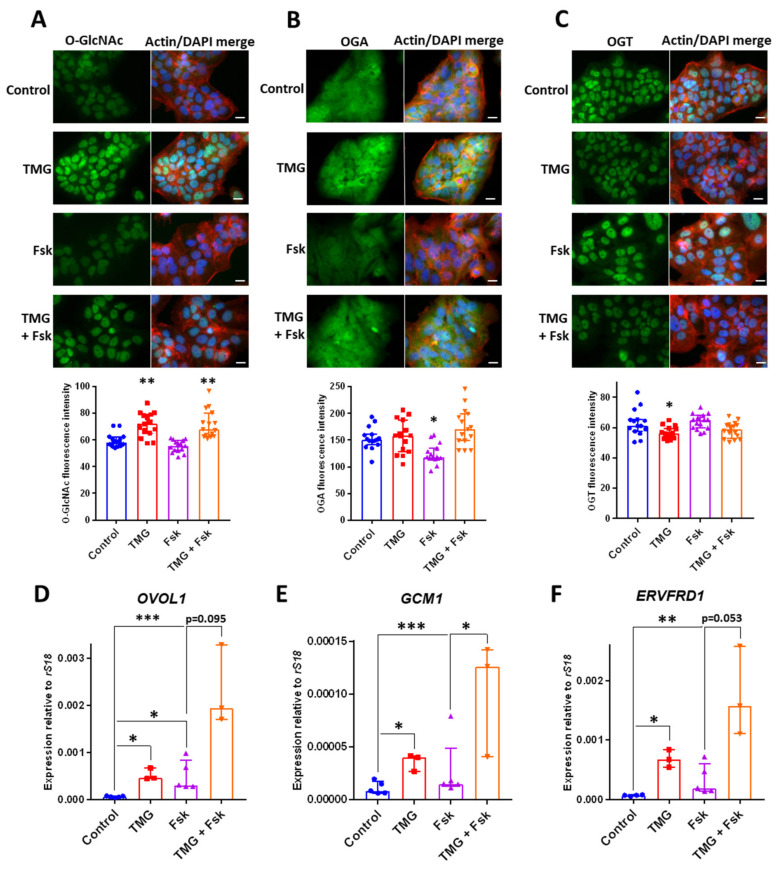
BeWo cells were treated for 24 h with DMSO (forskolin vehicle control), treated with 5 µM TMG and DMSO for 24 h, treated with 50 µM forskolin (Fsk) for 24 h, or treated with 5 µM TMG and 50 µM Fsk. (**A**–**C**) BeWo cells were immunostained with phalloidin (red), DAPI (blue) and (**A**) anti-O-GlcNAc antibody (green), or (**B**) anti-OGA antibody (green), or (**C**) anti-OGT antibody (green). Quantification of O-GlcNAc, OGA and OGT immunostaining mean fluorescence intensity (arbitrary units) is shown (*n* = 15 fields of view from three independent experiments, median ± IQR; * *p* < 0.05 ** *p* < 0.01 Control vs. TMG; Kruskal–Wallis). (**D**–**F**) qPCR was performed on BeWo cells to determine the expression of (**D**) OVOL1, (**E**) GCM1 and (**F**) ERVFRD1 (*n* = 3–5; median ± IQR; * *p* < 0.05; ** *p* < 0.01; *** *p* < 0.001; Kruskal–Wallis).

Human embryos pioneer endometrial invasion with multinuclear syncytiotrophoblast (STB) derived from TE cell fusion [38], and the BeWo human trophoblast cell line is a model for STB differentiation which can be induced by the adenylate cyclase activator forskolin [39]. TMG treatment of BeWo cells at 5 µM for 24 h (to mimic embryo treatments) increased O-GlcNAcylation levels and O-GlcNAc was localised to the nucleus with no clear nuclear envelope staining (Figure 4A). No change in levels of OGA immunostaining or nucleocytopasmic localisation was detected after TMG treatment (Figure 4B), whereas OGT levels were reduced but remained predominantly nuclear in localisation (Figure 4C). BeWo cells treated with forskolin for 24 h did not exhibit altered O-GlcNAc or OGT levels or localisation, although OGA levels were reduced (Figure 4A–C). Forskolin treatment alongside TMG did not affect O-GlcNAcylation levels (Figure 4A), but combined TMG and forskolin rescued OGA and OGT levels compared to forskolin alone and TMG alone, respectively (Figure 4B,C). 

We did not detect increased cell fusion after 24 h forskolin treatment (Figure 4A–C), mirroring previous studies [39,40]. TMG treatment led to increased expression of the STB-associated transcription factors *GCM1* and *OVOL1* to levels similar to those caused by treatment with forskolin (Figure 4C). Moreover, combined TMG and forskolin treatment led to further increases in *GCM1* and *OVOL1* levels (Figure 4C,D). *ERVW1* and *ERVFRD1* encode the cell fusion proteins syncytin-1 and -2, respectively, that mediate cell fusion to form STB. TMG induced upregulation of *ERVFRD1,* and combined TMG and forskolin treatment further upregulated *ERVFRD1* (Figure 4E). No effect of TMG on *ERVW1* expression was observed (data not shown).

## 4. Discussion

Differentiation of TE to invasive trophoblast is required for embryo implantation. This study reveals that protein O-GlcNAcylation is a potent driver of mouse TE differentiation to invasive TGC at implantation in vitro, as well as differentiation of BeWo to STB as a model of invasive human trophoblast at implantation. O-GlcNAcylation regulates the function of 1000+ proteins in line with metabolic and environmental status, contributing to the appropriate adaptation of cells to stressors [41]. Stress-induced differentiation has been implicated as a mechanism driving trophoblast invasion during implantation and placentation [9,10,11]; the present study evidences O-GlcNAcylation as a novel mediator of invasive trophoblast differentiation from TE that may play a role in the embryonic response to metabolic and environmental stress. Peri-implantation effects of ART procedures and maternal metabolic syndromes could, therefore, promote initial implantation, but this may be at the expense of long term placental function, which requires a balance of proliferative and invasive trophoblast populations that could be compromised by peri-implantation stress signalling [3].

To interrogate the effects of O-GlcNAcylation on implantation, we used the potent and highly selective OGA inhibitor, TMG [42], which has been widely used in in vitro and in vivo studies with no off-target effects observed [43]. TMG induced elevation of O-GlcNAcylation levels and kinetics is similar to that caused by cell stresses [19,34,44,45]. Moreover, TMG has been shown to increase O-GlcNAcylation in oocytes, leading to increased polyspermy [46], but it has not been used to treat pre-implantation embryos or trophoblast cells. The only previous attempt to elevate O-GlcNAcylation in pre-implantation embryos used the alternative OGA inhibitor PUGNAc [26], which is known to exhibit off-target effects [47]. In common with our study, Pantaleon et al. also used immunostaining with RL2 anti-O-GlcNAcylated protein antibody and showed nuclear and nuclear membrane localisation, as did more recent studies [27,28,29]. These localisations likely reflect a concentration of O-GlcNAcylated proteins in nuclear pore complexes [48], which play important roles in early embryogenesis [27,28], and among DNA-associated proteins such as transcription complexes and histones [49].

Localisation of OGT and OGA has not been reported previously in embryos, although OGT and OGA are classically nucleocytoplasmic enzymes with alternative splicing producing mitochondrial and lipid-droplet-associated isoforms, respectively [50,51]. Our observation of predominantly nuclear OGT in blastocysts and in BeWo trophoblasts chimes with reports from most cell types reported [48], and while nucleocytoplasmic shuttling has been observed in response to insulin and AMPK signalling [52,53], we did not detect altered subcellular localisation of OGT after TMG treatment. This may not be surprising as dynamic O-GlcNAcylation of Ser389 in OGT would be lost upon OGA inhibition, and this modification is coupled to nuclear import [54]. OGA is predominantly a cytosolic protein [55], as observed in untreated blastocysts; however, in BeWo cells, both nuclear and cytoplasmic localisation of OGA was observed regardless of TMG or forskolin treatment. We detected a striking translocation of OGA to the nucleus in blastocysts treated with TMG, but this effect was not observed in BeWo cells. Unlike OGT, mechanisms for nuclear translocation of OGA are not understood, but our data suggest that O-GlcNAcylation levels are coupled to its nuclear localisation in embryos. This could be driven by O-GlcNAcylation of OGA itself or by indirect mechanisms, and is suggestive of an attempt to dampen increased O-GlcNAcylation of nuclear proteins. Additionally, we demonstrated downregulation of *Ogt* and the rate-limiting HBP enzyme, *Gfpt1*, in blastocysts, and OGT in BeWo cells, in response to TMG, perhaps further suggesting negative feedback signals to constrain the HBP and O-GlcNAcylation.

Our in vitro model of implantation suggests that increased O-GlcNAcylation does not affect TE function with regard to attachment to endometrial epithelial cells. Activation of embryos through initial contact with endometrial epithelial cells between E4.5 and 5.5, prior to stable attachment, is required for blastocysts to go on to breach the epithelial layer [36], and we have previously reported that hyperosmotic stress-induced signalling through JNK can activate embryos in the absence of epithelial contact [10]. Here, TMG treatment during E4.5–5.5 also activated the embryos, suggesting that TMG-induced O-GlcNAcylation mimicked a stress response to promote breaching of the epithelium. E5.5 embryos activated by epithelial contact exhibit reduced expression of TE transcription factors *Cdx2*, *Gata2* and *Gata3* and increased expression of the TGC transcription factor *Hand1*, suggesting that activated TE initiates differentiation to TGC [36,37]. This pattern of downregulated TE transcription factors was observed at the transcript level in TMG-treated blastocysts and at the protein level for CDX2 and GATA3; however, *Hand1* transcript levels were not changed by TMG. The morphology of invasive embryonic cells after TMG treatment reflected TGC morphology previously observed, implying that downregulation of TE transcription factors drives TGC differentiation and the upregulation of *Hand1* is not required prior to E5.5. The absence of *Hand1* expression may be a signature of O-GlcNAcylation-induced TE differentiation and could precede the emergence of different trophoblast populations at implantation. O-GlcNAcylation has previously been shown to regulate chondrocyte, haematopoietic and corneal cell differentiation [33,56,57], amongst other cell types, indicating that that this mechanism is not specific to TE/trophoblast.

Using the BeWo human choriocarcinoma-derived trophoblast cell line to model STB differentiation at the epithelial phase of human embryo implantation [38,39], we showed that 24 h TMG treatment initiates the STB differentiation program. Conversely, our findings also show that STB differentiation is not accompanied by alteration to the global levels of O-GlcNAcylation. We demonstrated TMG-induced expression of early STB-associated transcription factors *OVOL1* and *GCM1,* which play distinct roles in suppressing proliferative gene expression and promoting the expression of genes that orchestrate syncytialisation, respectively [40,58]. *ERVW1* and *ERVFRD1* are two such genes, encoding syncytin-1 and -2, respectively, which mediate cell fusion [59], and here, we found that *ERVFRD1* was upregulated by TMG. Remarkably, TMG upregulated these genes to approximately the same levels as forskolin, which increases intracellular cyclic-AMP (cAMP) and induces trophoblast syncytialisation [39]. Moreover, TMG and forskolin may potentiate each other with respect to *OVOL1*, *GCM1* and *ERVFRD1* expression. Forskolin-induced BeWo cell fusion to form STB is observed only after treatment for 48h or longer [39,40], and correspondingly, we did not observe STB formation. These data could reflect O-GlcNAcylation of the cAMP-responsive protein kinase A (PKA), which has been identified to increase PKA activity and enhance the downstream signalling that promotes STB differentiation [60]. Alternatively, O-GlcNAcylation could regulate nuclear localisation of differentiation determinants in mouse and human trophoblast, as was seen for YAP1 in the mouse early TE [27], or chromatin accessibility through histone modification as observed during mouse trophoblast stem cell differentiation [61].

Our mouse embryo and human trophoblast data together implicate O-GlcNAcylation as a conserved mechanism that promotes invasive trophoblast differentiation, and this may reflect a stress response that unbalances the development of trophoblast populations at implantation. In vivo studies in mice and in vitro studies using human embryos and endometrial cultures are required to ascertain how peri-implantation stressors, whether due to ART procedures such as in vitro culture to blastocyst [62], or maternal metabolic disease such as diabetes [29], affect the proportion of proliferative and invasive trophoblast populations arising at this foundational time for establishment of an optimal maternal–foetal interface. Understanding implantation as a sensitive developmental window could lead to insights into treatments to improve ART success rates, but also to a better understanding of the great obstetric syndromes that are underpinned by placental dysfunction [63].

## Figures and Tables

**Table 1:** Details of antibodies used in this study.

Antibody (Clone/Catalogue Number)	Source
O-GlcNAc (RL2)	Biolegend (London, UK)
OGA (14711-1-AP)	Proteintech (Manchester, UK)
OGT (D1D8Q)	Cell Signaling Technologies (London, UK)
CDX2 (D11D10)	Cell Signaling Technologies
GATA3 (MAB6330)	R & D Systems (Abingdon, UK)

**Table 2:** Details of PCR primers used in this study.

Gene	Primers (5′–3′)
*Gfpt1*	CACCAATCGTGTCATCTTTCTGG GCAGTTCGTTTAATTCGGTGGAT
*Ogt*	TTGGCAATTAAACAGAATCCCCT GGCATGTCGATAATGCTCGAT
*Oga*	CATAGGATGTTTTGGCGAGAGAT CCTGGCGAAATAGCATAGATGAA
*Cdx2*	CAAGGACGTGAGCATGTATCC GTAACCACCGTAGTCCGGGTA
*Gata2*	CACCCCGCCGTATTGAATG CCTGCGAGTCGAGATGGTTG
*Gata3*	CTCGGCCATTCGTACATGGAA GGATACCTCTGCACCGTAGC
*Eomes*	GCGCATGTTTCCTTTCTTGAG GGTCGGCCAGAACCACTTC
*Elf5*	ACCGATCTGTTCAGCAATGAAG CGCTTGGTCCAGTATTCAGG
*Tfap2c*	ATCCCTCACCTCTCCTCTCC CCAGATGCGAGTAATGGTCGG
*Hand1*	CTACCAGTTACATCGCCTACTTG ACCACCATCCGTCTTTTTGAG
*Gapdh*	AGGTCGGTGTGAACGGATTTG GGGGTCGTTGATGGCAACA
*OVOL1*	TGAACCGCCACATGAAGTGTC GACGTGTCTCTTGAGGTCGAA
*GCM1*	CCAATTCCAGCGGGTAATCTT GGTGAATGGTATGCAGGAGAC
*ERVFRD1*	ACCGCCATCCTGATTTCCC GAGGCTGGATAAGCTGCTCC
*S18*	CGGCTACCACATCCAAGGAA GCTGGAATTACCGCGGCT
